# Teaching the Virtual Brain

**DOI:** 10.1007/s10278-022-00652-5

**Published:** 2022-05-23

**Authors:** Javier Hernández-Aceituno, Rafael Arnay, Guadalberto Hernández, Laura Ezama, Niels Janssen

**Affiliations:** 1 Departamento de Ingeniería Informática y de Sistemas, Universidad de La Laguna, Avda. Astrofísico Fco. Sánchez s/n, La Laguna, 38204 Canary Islands Spain; 2 Departamento de Fisiología, Universidad de La Laguna, Campus de Ofra s/n, La Laguna, 38071 Canary Islands Spain; 3 Departamento de Psicología Cognitiva, Social y Organizacional, Instituto de Tecnologías Biomédicas e Instituto Universitario de Neurociencia, Universidad de La Laguna, Campus de Ofra s/n, La Laguna, 38071 Canary Islands Spain

**Keywords:** Brain, Virtual reality, Education, Brain exploration

## Abstract

As a complex three-dimensional organ, the inside of a human brain is difficult to properly visualize. Magnetic Resonance Imaging provides an accurate model of the brain of a patient, but its medical or educational analysis as a set of flat slices is not enough to fully grasp its internal structure. A virtual reality application has been developed to generate a complete three-dimensional model based on MRI data, which users can explore internally through random planar cuts and color cluster isolation. An indexed vertex triangulation algorithm has been designed to efficiently display large amounts of complex three-dimensional vertex clusters in simple mobile devices. Feedback from students suggests that the resulting application satisfactorily complements theoretical lectures, as virtual reality allows them to better observe different structures within the human brain.

## Introduction

The human brain is a complex three-dimensional object that resides inside our cranium. Our understanding of the brain has increased enormously with the advent of recent neuro-imaging techniques such as Magnetic Resonance Imaging (MRI) [1, 2]. The MR technique creates 3D matrices that contain signal intensity values determined by the specific magnetic properties of the tissues in particular locations of the brain. The most commonly used method for visualizing these 3D matrices relies on displaying 3D versions of the images on a 2D computer monitor [3].

This standard method of visualization is used for diagnosis and prognosis in clinical contexts, to study brain function in research contexts, and to study the underlying principles of neuro-anatomy and physiology in educational contexts. However, despite its widespread use, this visualization method is problematic because the 2D images do not preserve accurate depth information, and do not permit easy interaction. Consequently, improving methods for visualization may be beneficial to this wide range of contexts.

This work presents a mobile application to expedite the teaching of brain anatomy by visualizing MR images and the different brain structures using Virtual Reality (VR). Although techniques for VR have been around for years [4], recent technological advancements in small-scale computing have made VR accessible to the masses. Specifically, modern mobile phones now possess sufficient computing power to render a fully interactive VR experience [5]. Our particular VR setup relied on a low-cost solution: a standard Android phone (LG Nexus 5x with Android 6.0 Marshmallow) combined with a VR headset [6]. We used the Unity3D platform as a local rendering engine [7].

### Related Work

Visualizing the human brain using VR is not new; early approaches date back to 2001. The advantages of using VR are that it preserves accurate depth information, and that it potentially allows for a natural interaction with the visualized object. Zhang et al. [8] displayed diffusion tensor magnetic resonance images using a virtual environment, which consisted of an $8\times 8\times 8$ foot cube with rear-projected front and side walls and a front-projected floor; in this setup, the user wore a pair of LCD shutter glasses which supported stereo-viewing. Ten years later, Cheng et al. [9] presented a virtual reality visualization method which used a two-screen immersive projection system, required passive projection and glasses for a stereoscopic 3-D effect, and involved an Intersense IS-900 6-DOF tracking system with head-tracker and wand.

As technology evolved, VR systems were integrated into ever smaller devices, such as mobile phones and virtual reality glasses. Kosch et al. [10] used VR as input stimulus to display real time, three-dimensional measurements using a brain–computer interface. Soeiro et al. [11] proposed a mobile application which used virtual and augmented reality to display the human brain and allowed the user to show or hide complete regions. Prior applications primarily convert the MR images to surface meshes and do not permit the examination of the internal structure of the brain. The application presented in this paper allows the user to make arbitrary cuts that reveal the underlying brain structure directly from the MR image, and to generate voxel clusters from arbitrary seed points, with high detail and fidelity to the original data.

The benefits of the educational application of VR systems have been extensively studied before: Schloss et al. [12] included audio to narrate information as part of guided VR neuroanatomy tours, and Stepan et al. [13] used computed tomography and highlighted the ventricular system and cerebral vasculature to create a focused interactive model. Several different technologies have also been used to produce educational VR experiences, including the origin of the presented anatomical data (magnetic resonance [14], dissection [15]), the hardware which runs the applications (HTC Vive [16], Dextrobeam [17]), or the software which presents the simulation (virtual presentations [18], fully interactive applications [19]). All works however agree that allowing students to study anatomical models in an interactive virtual environment greatly improves their understanding of the matter. The presented work builds upon this concept and introduces a new slicing feature that allows students to explore the human brain in greater depth.

This paper is organized as follows: “Educational Application” explains the educational goals of the presented work; “Virtual Brain in the Classroom” then details the protocol used to introduce students to the developed application, which is then described in “Material and Methods”, along with the actions a user can perform in it; “Calculation” then presents the algorithms upon which the application is based; “Results and Discussion” studies the user feedback regarding the presented work; finally, “Conclusions” provides a conclusion on the usefulness of the application.

### Educational Application

The VRBrain application will be used in two separate courses. The first course, Biological Psychology (BP), forms part of the Master degree of Biomedicine at the University of La Laguna. The course consists of 3 ECTS credits (European Credit Transfer and Accumulation System) and takes place across a period of 3 weeks in daily 2 hour sessions. The course is typically taken by students that intend to pursue a doctoral degree in the PhD program in Medicine where a Master’s degree is required. The focus of the course is on how the various human cognitive and behavioral skills are implemented in the brain and how they are affected by disease.

The course is divided into two main sections: One section that examines these issues using the Magnetic Resonance Imaging (MRI) technique and one that examines these issues using the Electro-Encephalography (EEG) technique. These two techniques permit insight into brain structure and function and allow for the examination of the brain under pathological circumstances. The defined skills that students are required to have mastered at the completion of the course are the following:

- Understand basic anatomical organization of the human brain
- Understand how brain pathology can affect basic functions like memory and language
- Understand how brain pathology can affect basic functions like attention and perception

The first section of the course explains the MRI technique and details how this tool has been used to understand basic functions like memory and language. The course also examines how brain pathology that affects these functions can be elucidated using MRI. For example, it is commonly known that abnormal aging and Alzheimer’s Disease are related to memory problems and MRI has played a pivotal role in showing that such memory dysfunctions are associated with reductions in gray matter volume that start in a specific brain region called the hippocampal formation. In addition, another brain pathology called cerebral stroke sometimes leads to a specific language problem called Broca’s aphasia, and MRI has shown that such problems are associated with lesions in a part of the brain called the left inferior frontal gyrus.

In order to understand these issues, students should learn the brain’s division into its main structures (the cerebral lobes, the ventricles, the meninges, etc.), as well as know some of the finer details of the organization of the brain (e.g., the parcellation of the cerebral cortex into its main areas, frontal lobe, temporal lobe, etc.). The BP course is then focused on the hypothesized function of these different parts of the brain and the role they may play in pathology.

In the second part of the course, the EEG technique will be used to address similar issues in the context of attention and perception. However, given the limitations of the EEG technique to yield images of the internal structure of the brain, the VRBrain application will be primarily used in the first section of the course. The specific structure of this first section of the course is as follows:

- Basic concepts in functional Magnetic Resonance Imaging (fMRI): Physical basis, biological basis, hands-on experience—7 hours
- Learning and Memory: Aging, Alzheimer’s Disease—4 hours
- Language: Language disorders and the brain, language lateralization, recent evidence—4 hours

The section on fMRI and the hands-on experience are intended to use the VRBrain application.

The second course in which we intend to use the VRBrain application is the Undergraduate Thesis Projects (UTP) which are mandatory under the Spanish university system. The UTP is a 6 ECTS credits course which takes place in the second semester of the fourth year in the Psychology Degree at the University of La Laguna. The aim of this course is to allow for students to develop their own interest on a given research topic. They work together with a professor to establish a research idea and then do autonomous work to perform the research and write the thesis.

Given the large number of final year students in the Psychology degree that need to do the UTP, the students need to choose from a number of research themes that are proposed by professors in the Psychology department that are teachers in the UTP course. Some of these research themes are:

- Educational Psychology
- Personality Psychology: Mindfulness, stress, anxiety
- Basic Psychology: Neuro-imaging; Neuro-anatomy; Language; Memory

The research theme in which the VRBrain will be applied is the research theme related to neuro-imaging and neuro-anatomy. The research topics in this research theme are related to investigation of the brain and pathology, and as such, it is useful if students understand the basic neuro-anatomy of the brain. Here the VRBrain application will be very useful.

### Virtual Brain in the Classroom

As we pointed out in “Educational Application”, the main goal of the application is to increase the understanding of neuro-anatomy in students at both the undergraduate and master’s degree levels. In the classroom setting we will have implemented the following protocol in the usage of the VRBrain application. The duration of the entire protocol is around 2 hours.

First, we will divide students in the class into small groups of 3 to 4 people. This will ensure that the application is used by everyone including those that do not have an Android device. In addition, given that the number of VR headsets is limited, this will also ensure that everyone will be able to use the application.

Second, given the complexity of the application, we will first give students the ability to get familiar with the application. This means they are free to start up the application and explore the various menus using the Bluetooth controller. In this next section they are required to perform a small quiz in which we present a number of targeted questions that require finding and understanding basic neuro-anatomy of the human brain. This mainly relies on the first functionality of the application (see “Material and Methods”). For example, we have questions such as:

- Describe in anatomical terms the location of the human hippocampus in relation to the amygdala.
- Does Broca’s region lie in the frontal or temporal lobe?
- What is the main function of the occipital lobe?

Answering these questions relies on a 3D understanding of the brain, as well as having read the information that appears in the textbox when a given structure is highlighted within the VRBrain application.

In addition, we also ask students to examine the internal structure of the brain using the second functionality of the application (see “Brain Slicing” in “Material and Methods” as follows). Within this functionality we will ask questions such as

- Estimate the distance from the superior part of the brain to the lateral ventricle in centimeters.
- Find the hippocampal area by slicing the brain in coronal slices
- Find the corpus callosum by slicing the brain in sagittal slices

Answering these questions requires inspection of the internal structure of the brain which is implemented in the VRBrain application. We hope that by studying these questions in the classroom the students develop further insight into the 3D structure of the brain and improve their understanding of human neuro-anatomy. This will then in turn improve their understanding of the larger topics related to brain function and pathology in the respective courses.

## Material and Methods

**Fig. 1:**
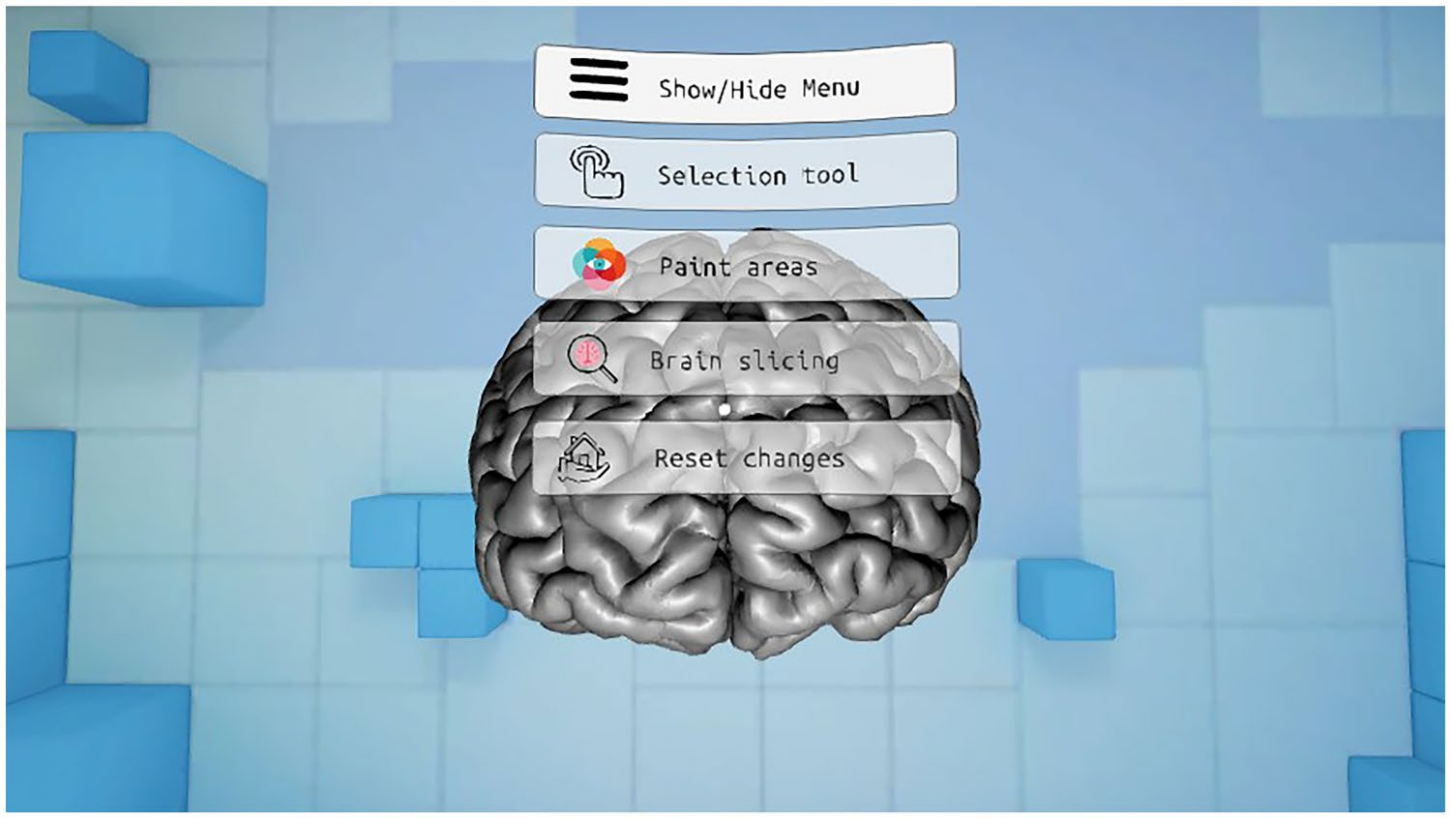
Main menu of the Virtual Brain application

The application is divided into two main parts. First, a series of functionalities have been implemented to color and highlight different parts of the brain and show information about them. Second, a functionality has been developed in which the user can make cuts in the brain in orthogonal planes to the point of view. In this application, the user can interact with the menu entries by looking directly at them for a short period of time. The analog stick is used to rotate the virtual representation of the brain. Figure 1 shows the main menu of the presented application.

### Brain Slicing

Brain Slicing is a functionality to study the internal anatomy of the brain. The user can rotate the view around the virtual brain and perform cuts in an orthogonal plane to the point of view. The virtual representation of the brain is made from MRI data that must be preprocessed to generate both the internal information of the brain and the cortical surface. In the next sections, both the preprocessing step and the calculations necessary to carry out the cuts both in the cortex and in the internal representation of the brain are detailed.

#### Preprocessing

In the first step of the process, the raw MRI data that is obtained from a patient scan is stored in the standard Digital Imaging and Communications in Medicine (DICOM) format. As this image format generally does not permit easy manipulation, all DICOM images were transformed into a data format called *Neuroimaging Informatics Technology Initiative* (NIfTI [20]). The NIfTI file format includes the affine coordinate definitions relating voxel index to spatial location and codes to indicate the spatial and temporal order of the captured brain image slices. Although the developed application accepts files of any size, the default resolution of the examples in the presented work is $256\times 256\times 128$ voxels.

This NIfTI file is first processed through the *BrainSuite* cortical surface identification tool, which produces a three-dimensional vertex mesh of the brain cortex [21]. This step is necessary because a raw representation of the voxels of a NIfTI file most commonly offers a dull and unrealistic appearance. However, in order to properly display any segmentation of a brain, both the cortical and inner data are required; therefore, both the cortex mesh and the vortex data matrix are loaded onto the visualization program.

**Fig. 2:**
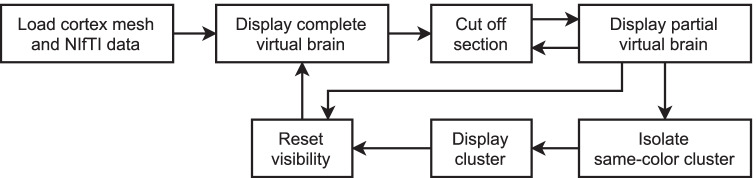
Flowchart of the brain slicing functionality

The user can then freely rotate the view around the virtual representation of the brain, and they may choose to perform three different actions: cutting off a section by defining an intersection plane, isolating a specific same-colored region of the brain, and restoring the whole brain to its original state. “Calculation” explains how these operations are executed. The flowchart of the presented application is displayed in Fig. 2.

**Fig. 3:**
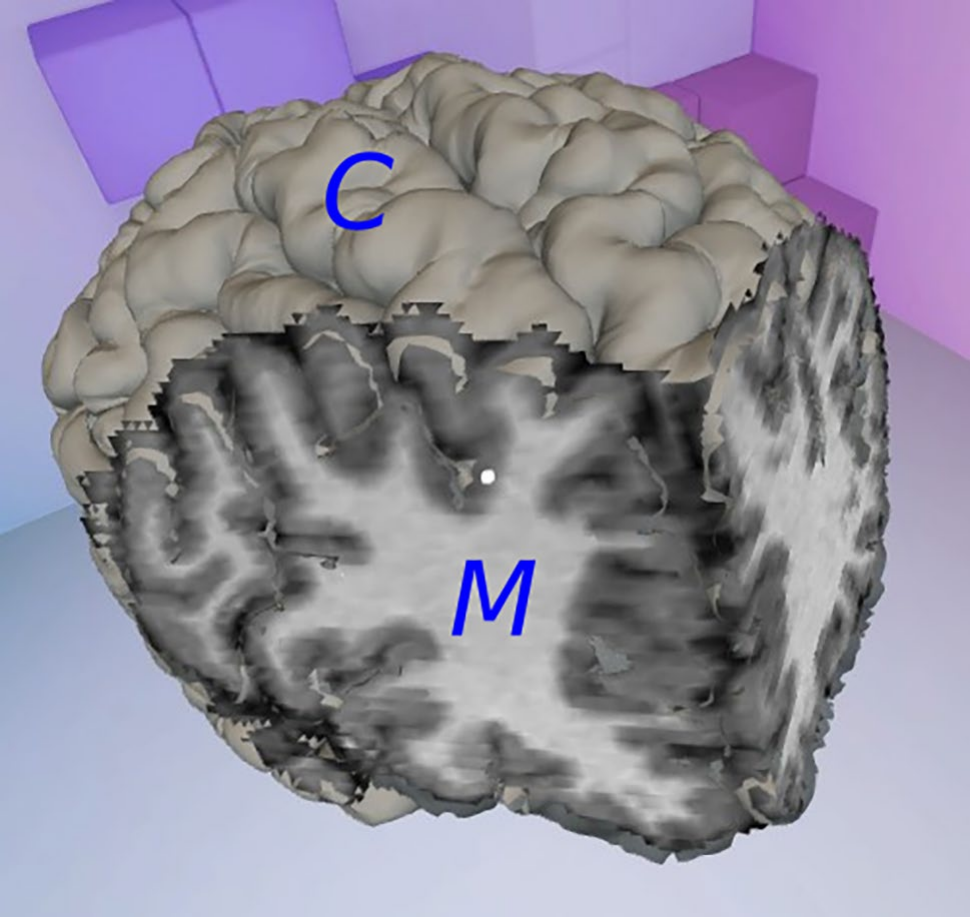
Cortex (*C*) and inner mesh (*M*) of the virtual brain

A second mesh is created when a cut or an isolation is produced, showing the faces inside the brain that become visible (Fig. 3). A three-dimensional mesh normally contains the locations of all vertices, some metadata regarding their normal vectors, colors and/or texture mapping, and a list of triangles, which describe the connections between vertices in order to form visible faces.

The vertices of the inner brain mesh are the centers of all the voxels provided by the NIfTI file, and their color is the gray level defined by their fMRI value. To generate a clearer image, shading is not taken into account, so the normal vectors of the vertices are unnecessary and ignored. Visible faces only appear at the edge between a visible region of the brain and a hidden one, so the triangles of the inner mesh are calculated every time the user produces a cut or an isolation, as explained in “Calculation”.

## Calculation

**Fig. 4:**
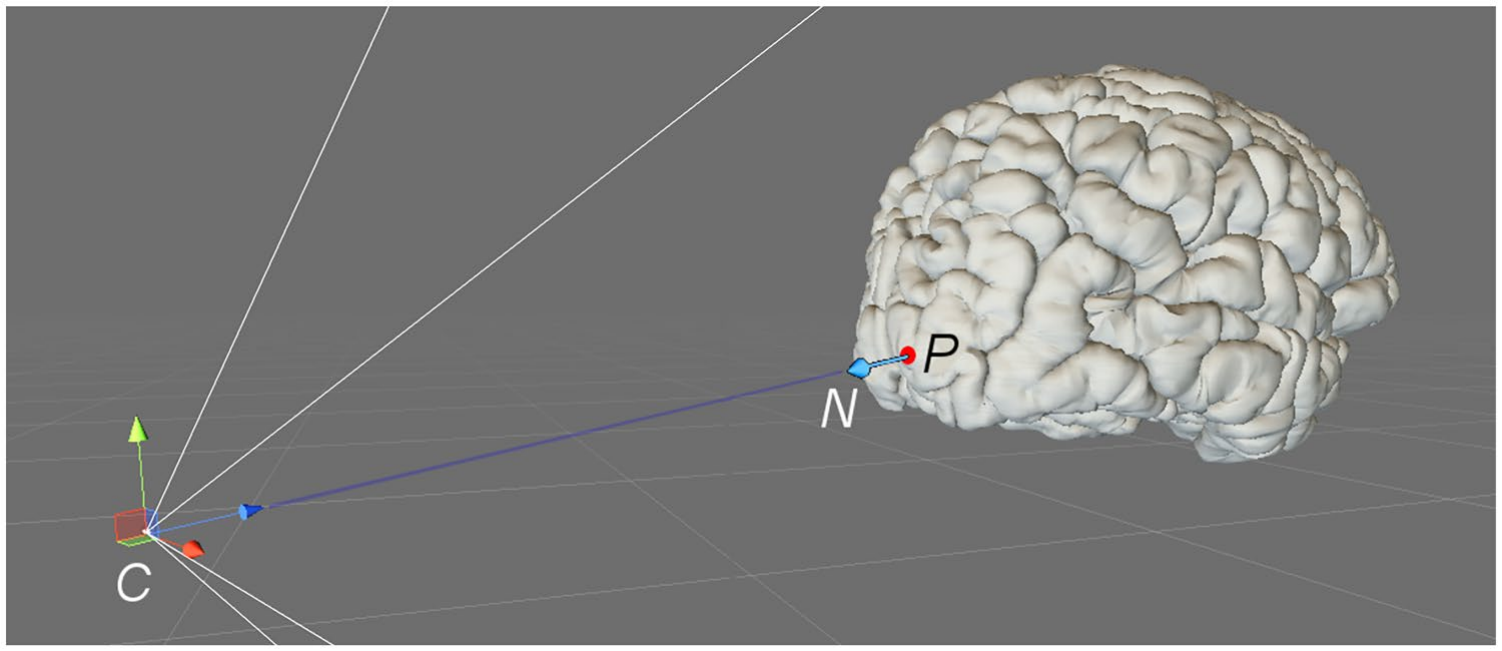
Plane point (*P*) and normal vector (*N*), as defined by the user camera (*C*)

To select a plane with which to cut off a portion of the brain, a point in space *P* and a normal vector *N* are needed. Both elements are extracted from the point of view of the user, relative to the center of the brain: the orientation vector of the camera in the scene equals $-N$, while *P* is located at the center of the closest active brain voxel on which the user focuses their gaze (Fig. 4). Point *P* can also define a seed voxel to isolate a same-colored region of the brain.

Once the user selects a cut plane, all voxels of the virtual brain are then classified according to their relative position. Let *Q* be the center of a voxel, its signed distance to the plane is calculated as the scalar product $N\cdot \left( P-Q\right)$; if this value is negative, the voxel is located between the cut plane and the user camera and must be removed.

**Fig. 5:**
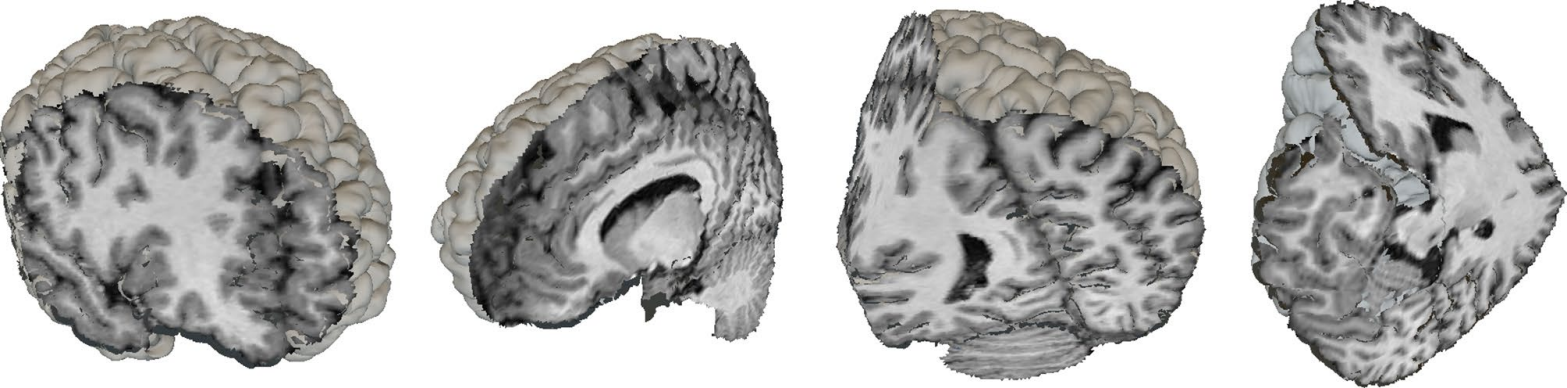
Examples of arbitrary plane cuts

The cortex mesh is also updated every time a brain section is cut off. Removing its vertices is not necessary, since they will not be visible unless they are referenced by at least one triangle. Therefore, when the user defines a cut plane, only the list of triangles of the mesh is updated by removing every triangle which contains one or more vertices located between the plane and the user camera (Fig. 5).

**Fig. 6:**
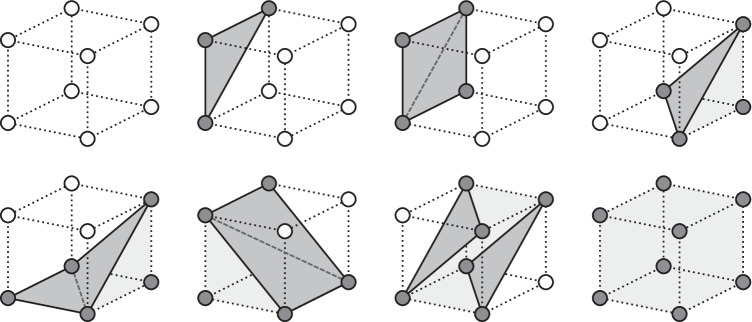
Examples of triangulation neighborhoods, where white vertices are invisible and dark vertices are visible

To calculate the triangles of the inner brain mesh, all $2\!\times \!2\!\times \!2$ vertex neighborhoods of the inner brain mesh are studied individually. Since visible faces only form at the edge between visible and invisible regions of the brain, triangles will only connect visible vertices that are close to at least one hidden vertex (Fig. 6). A neighborhood contains only 8 vertices, which can either be visible or invisible, so the amount of possible face combinations per neighborhood is $2^8$. To decrease calculation time during execution, these combinations are precalculated as shown in Algorithm 1 and reused for each vertex neighborhood of the virtual brain.

**Figure:**
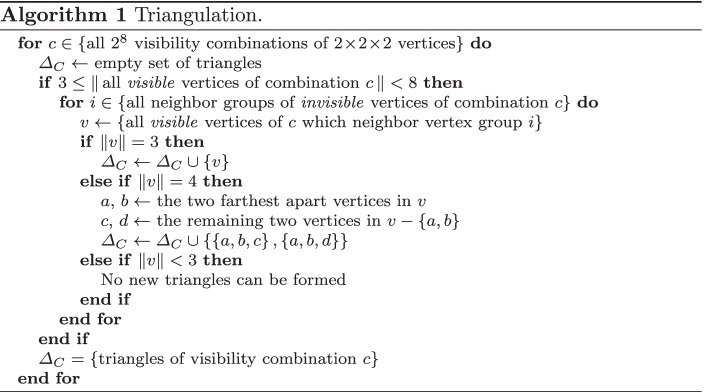

**Fig. 7:**
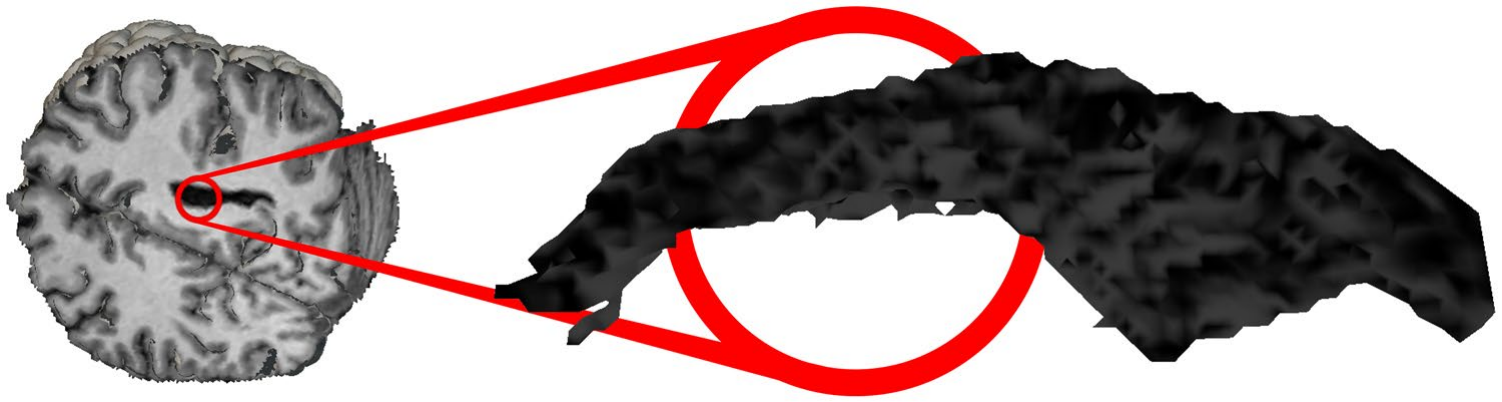
Example of a cluster of same-colored voxels

Once the inside of the brain becomes visible, the user can select an isolation seed by focusing their gaze on a specific brain voxel. When this happens, every voxel in the brain is marked as invisible; then, the visibility of the seed and every neighboring voxel with a similar enough gray level, given a threshold, is restored as shown in Algorithm 2. The result is a single cluster of voxels of similar color (Fig. 7). The cortex is completely hidden in this situation, so that the cluster can be seen clearly, and the triangulation process described in Algorithm 1 creates a visible mesh around the isolated voxels.

**Figure:**
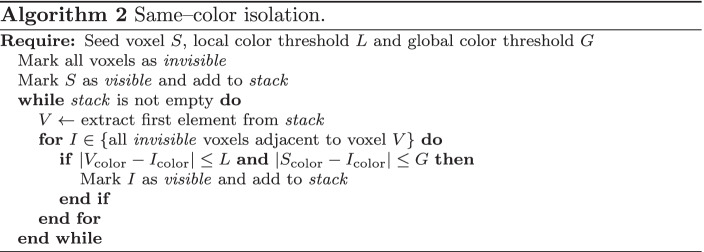

Finally, the restoration action simply returns all voxels back to their original state and resets the triangles of the cortex mesh.

### Paint Areas and Selection Tool

The application also includes functionalities to paint areas of the brain, to highlight both internal and external structures and to display information about them. Virtual reality is used so that the user can better appreciate the shape and spatial arrangement of these structures within the brain.

#### Selection Tool

**Fig. 8:**
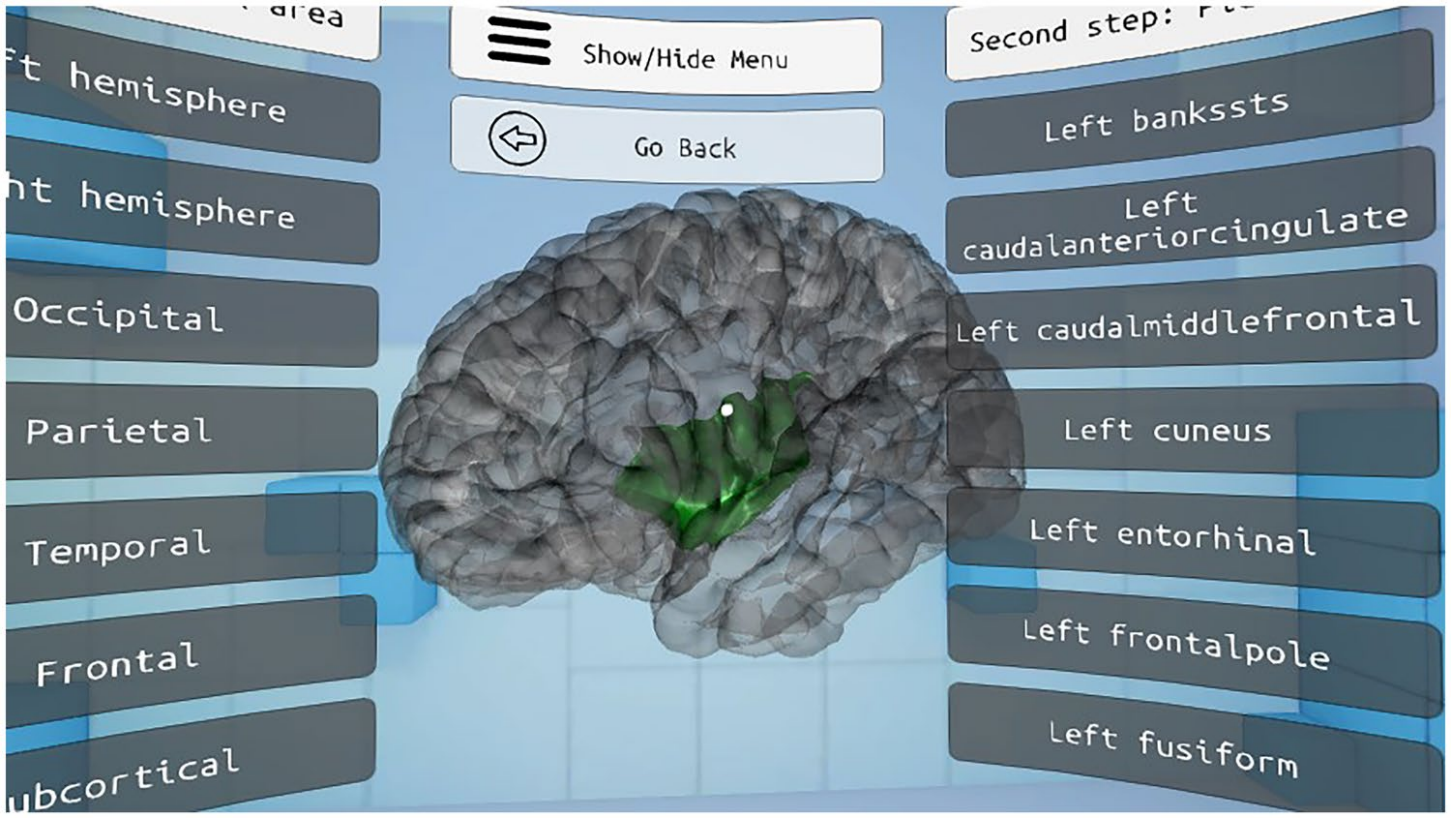
Selected area of the brain shown in green inside a semitransparent brain

**Fig. 9:**
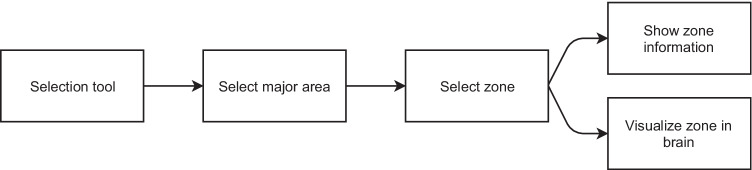
Flowchart of the Section tool

**Fig. 10:**
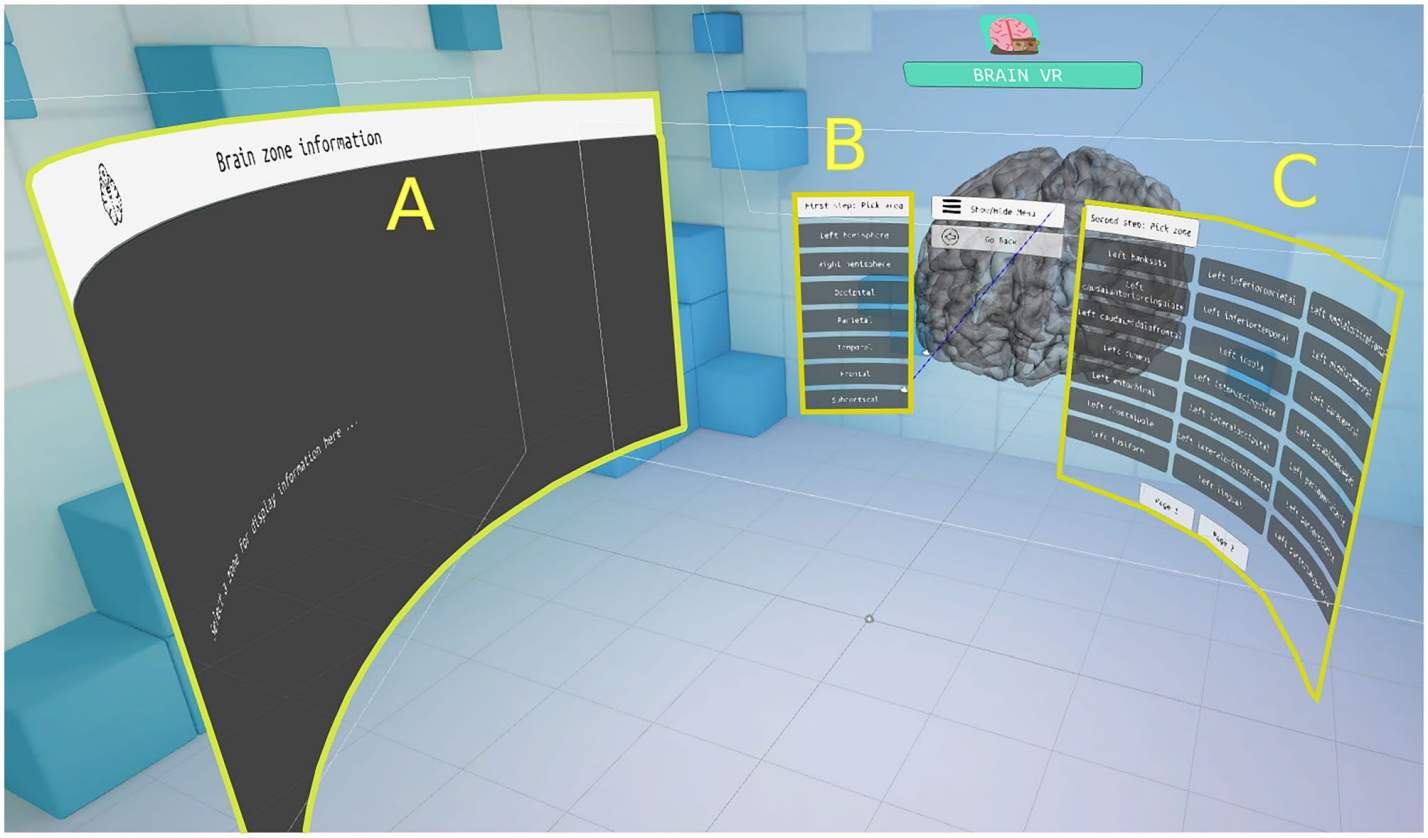
Selection tool interface: area where information is displayed (**A**), zone selection menu (**B**) and area selection menu (**C**)

The selection tool allows the user to select a part of the brain and visualize its shape and location; information about its functions is also displayed. To select a region, the user must first select the zone in which it is located, in a menu on the left side. Then, a menu is displayed to the right of the user, displaying the structures which the selected zone contains. Finally, the selected area is shown in green color inside a semitransparent brain, as seen in Fig. 8. Figure 9 shows a flowchart of this functionality and Fig. 10 shows the different interfaces which the user can access in the selection tool environment.

#### Paint Areas

**Fig. 11:**
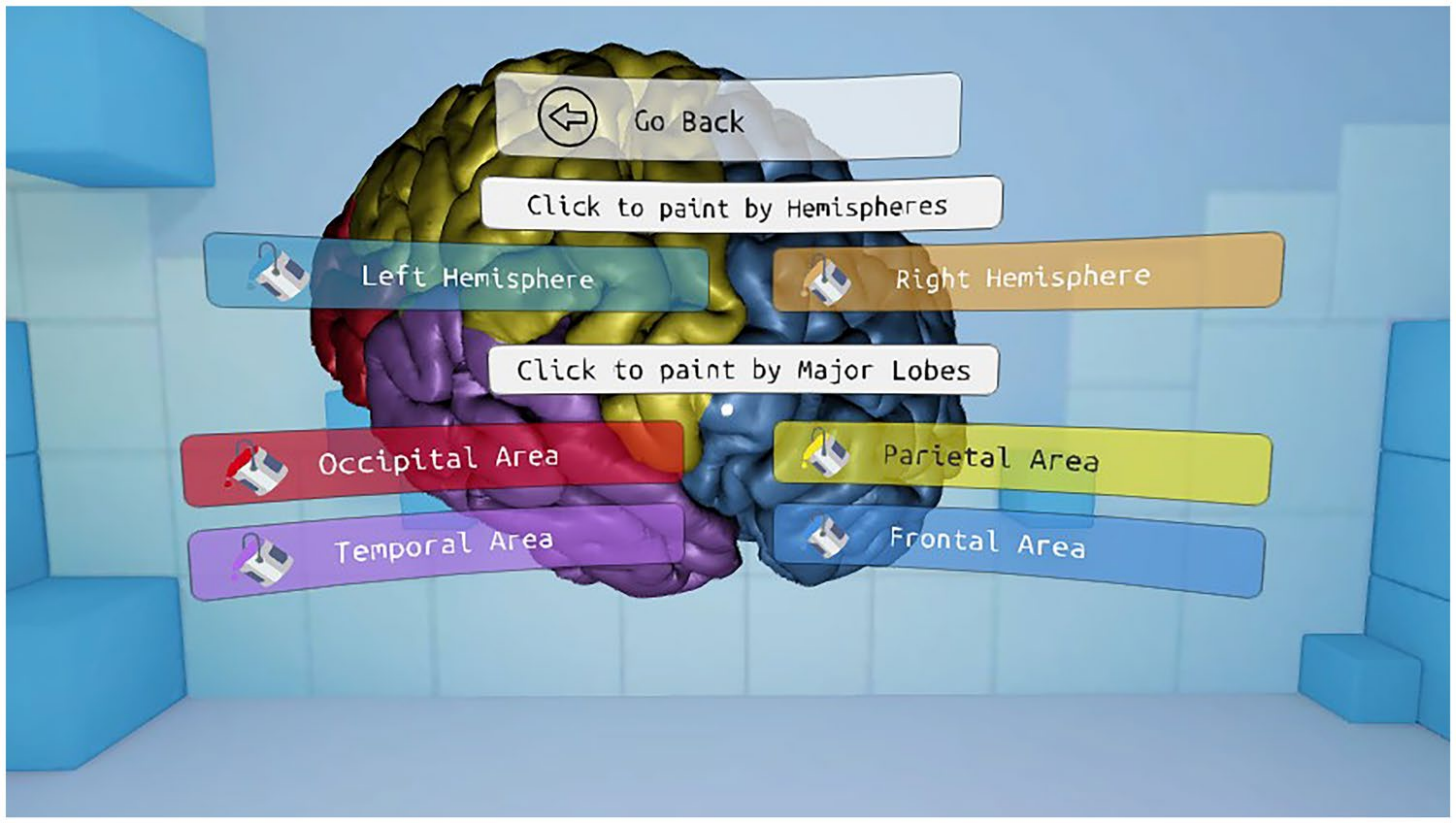
Interface of the Paint areas functionality

**Fig. 12:**
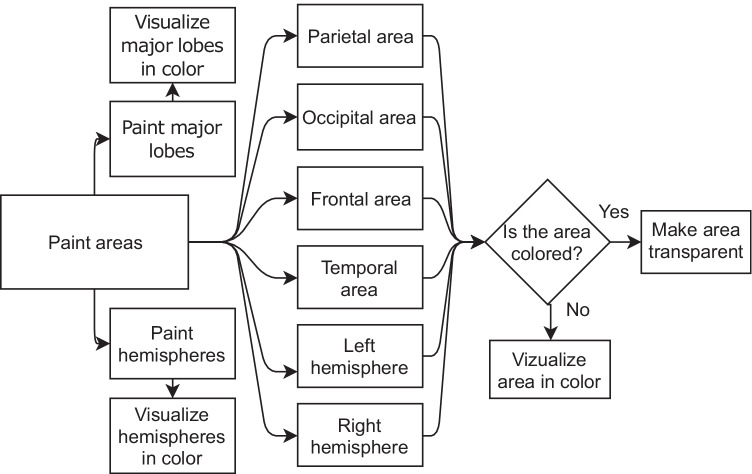
Flowchart of the Paint areas functionality

The functionality to paint areas allows the user to visualize different cortical areas of the brain in color. The interface is composed of two main buttons that activate two different color schemes: one to visualize the hemispheres and the other to show the major lobes. In addition, the user has access to buttons to color each area individually. If the area is already colored, clicking the button turns it semi-transparent, so that the internal structure of the brain is exposed. Figures 11 and 12 show the flowchart and the interface of this functionality, respectively.

## Results and Discussion

The developed VR application was presented to 32 students, ages between 19 and 37 years old, and their opinion on its performance and usefulness was collected in an anonymous five point Likert scale satisfaction questionnaire [22]. Table 1 shows the mean, standard deviation and $95\%$ confidence interval of the questionnaire results.

These results show that students have found the application to be helpful in their learning process, as represented by their opinion on the “Paint areas” and “Selection tool” functionalities (mean above 4.44). This is in line with previous works, which found that the usage of virtual reality significantly improves test grades [17], since it helps students to better understand the three-dimensional structures of the human brain [18], also improving their satisfaction and decreasing their reluctance to learn neuroanatomy [14].

The “Brain slicing” option scored only an average 3.33 out of 5. This tool was not originally designed as an educational device, but for medical experts to explore the brain of a real patient and search for anomalies; as such, students found it too complicated to use and not instructive enough. Further iterations of this work may attempt to adapt this option to increase its formative potential.

Students also mostly agree that the presented application should be used in following courses and find its usage easy and intuitive, the “Brain slicing” option again scoring lower than the other functionalities.

**Table 1:** Results of the usability questionnaire

**Statement**	**Mean**	**Std**	**95% CI**
“Paint areas” has improved my understanding of the location and shape of the hemispheres	4.69	0.47	[4.52 - 4.85]
“Paint areas” has improved my understanding of the location and shape of the major lobes	4.62	0.61	[4.41 - 4.83]
“Selection tool” helped me understand the location and shape of both cortical and subcortical areas	4.65	0.60	[4.45 - 4.86]
“Selection tool” helped me understand the difference between the cortical and subcortical areas	4.44	0.67	[4.20 - 4.67]
“Brain slicing” has allowed me to understand the relationship between white matter and gray matter	3.31	1.35	[2.84 - 3.78]
I find the possibility offered by “Brain Slicing” to make arbitrary cuts in the brain volume useful	4.09	1.33	[3.63 - 4.55]
VR has improved my perception the relative size of the different structures within the brain	4.53	0.80	[4.25 - 4.81]
Do you think that the Paint areas functionality should continue to be used in the following courses?	4.84	0.37	[4.71 - 4.97]
Do you think that the Selection tool should continue to be used in the following courses?	4.84	0.45	[4.69 - 5.00]
Do you think that the Brain slicing tool should continue to be used in the following courses?	4.28	1.11	[3.89 - 4.67]
The presented tools have increased my interest in this subject	4.38	0.94	[4.05 - 4.70]
The interface of the Paint areas functionality is intuitive and easy to use	4.66	0.54	[4.47 - 4.84]
The interface of the Selection tool functionality is intuitive and easy to use	4.59	0.56	[4.40 - 4.79]
The interface of the Brain Slicing functionality is intuitive and easy to use	3.44	1.32	[2.98 - 3.89]
The presented tools have reduced the time required to learn the brain anatomy	4.25	0.72	[4.00 - 4.50]
The presented tools adequately complement the theoretical explanations provided by the teacher	4.47	0.62	[4.25 - 4.68]
I do not find the Paint areas functionality to be unnecessarily complex	4.06	1.37	[3.59 - 4.53]
I do not find the Selection tool functionality to be unnecessarily complex	4.03	1.38	[3.55 - 4.51]
I do not find the Brain slicing functionality to be unnecessarily complex	3.15	1.44	[2.66 - 3.65]

## Conclusions

A VR brain exploration application has been developed as a medical and educational tool. The presented system builds a three-dimensional brain model from MRI data and a basic cortex model, then allows the user to cut slices off in order to study its inside and isolate vertex clusters by color. Students can also highlight and analyze different areas of the brain in order to complement their anatomical knowledge.

A satisfaction questionnaire showed very positive feedback from the students who tested the application, who claim that the educational side of the presented work was very useful to them, as it helped them better understand the theoretical explanations provided by the teacher.

The results obtained in the present study fit well with previous works, such as [13, 14, 16, 17], or [19], but the implemented virtual experience allowed for a greater degree of interaction than Schloss et al. [12], Lopez et al. [18] and de Faria et al. [15], due to its unique vertex triangulation algorithm and novel exploration tools which may also be used for medical and non-educational purposes. Based on student feedback, further iterations of the presented work will improve some of the presented features to increase their approachability in an academic environment.

## Data Availability

All collected data is included as part of the presented work

## Code Availability

All developed code is available at github.com/jhaceituno/brain3D.
